# Questionable Efficacy of Therapeutic Antibodies in the Treatment of Anthrax

**DOI:** 10.1128/mSphere.00282-19

**Published:** 2019-06-19

**Authors:** Jean-Nicolas Tournier, Clémence Rougeaux, Fabrice V. Biot, Pierre L. Goossens

**Affiliations:** a Institut de Recherche Biomédicale des Armées, Bacteriology, Anti-infectious Biotherapies, and Immunity Unit, Brétigny-sur-Orge, France; b Institut Pasteur, Viral Genomics and Vaccination Unit, CNRS UMR-3569, Paris, France; c National Reference Center for Anthrax (CNR-LE Charbon), Brétigny-sur-Orge, France; d Ecole du Val-de-Grâce, Paris, France; e Institut Pasteur, Yersinia Unit, Paris, France; Antimicrobial Development Specialists, LLC

**Keywords:** anthrax, antitoxins, monoclonal antibodies, protective antigen, toxins

## Abstract

Inhalational anthrax caused by Bacillus anthracis, a spore-forming Gram-positive bacterium, is a highly lethal infection. Antibodies targeting the protective antigen (PA) binding component of the toxins have recently been authorized as an adjunct to antibiotics, although no conclusive evidence demonstrates that anthrax antitoxin therapy has any significant benefit. We discuss here the rational basis of anti-PA development regarding the pathogenesis of the disease. We argue that inductive reasoning may induce therapeutic bias. We identified anthrax animal model analysis as another bias. Further studies are needed to assess the benefit of anti-PA antibodies in the treatment of inhalational anthrax, while a clearer consensus should be established around what evidence should be proven in an anthrax model.

## Abstract (precis)

Inhalational anthrax caused by Bacillus anthracis, a spore-forming Gram-positive bacterium, is a highly lethal infection. Antibodies targeting the protective antigen (PA) binding component of the toxins have recently been authorized as an adjunct to antibiotics, although no conclusive evidence demonstrates that anthrax antitoxin therapy has any significant benefit. We discuss here the rational basis of anti-PA development regarding the pathogenesis of the disease.

## OPINION/HYPOTHESIS

Natural inhalational anthrax is a seldom-seen disease. It came into the limelight after the bioterrorist attacks in the fall of 2001, when dried anthrax spores were sent through the mailing system in the United States. During this outbreak, 5 of the 11 patients died despite supportive therapy and antibiotic administration (1). The 2001 inhalational anthrax patient cohort had the best historical survival rate, although no specific therapeutic was available at that time besides antibiotics (2). In 2004, the Project BioShield Act (3) was launched to develop specific medical countermeasures. It financed the U.S. strategic national stockpile, which stores products in case of a public health emergency. So far, its large funding has enabled further research into targeting the toxins via vaccines and therapeutic antibodies.

Fifteen years later, three anthrax antitoxins have been approved by the U.S. Food and Drug Administration (FDA): two of them are monoclonal antibodies (raxibacumab and obiltoxaximab), and the third is human polyclonal purified IgG from vaccinated humans, intravenous anthrax immune globulin (AIG-IV). These products have been stockpiled with the support of Project BioShield, for a total cost not publicly released but estimated to be around several hundred millions of dollars according to the last report in 2014 (4). In the wake of FDA authorizations, the Centers for Disease Control and Prevention (CDC) updated their guidelines for anthrax postexposure prophylaxis and treatment, recommending that antitoxin should be added to antibiotic therapy for any patient suspected of a systemic anthrax infection. Recent closer examination of available clinical and preclinical data has cast doubt upon the beneficial effect afforded by the antibiotics and antitoxin combination in preclinical models (5, 6). Further clinical data collected on the particular form of injectional anthrax during the drug user outbreak in the United Kingdom indicated that death rates did not differ significantly between AIG-IV recipients and nonrecipients (33% versus 21%, with 43 patients included in the study [15 AIG-IV recipients and 28 nonrecipients]), suggesting no benefit of the therapeutics (7). Three patients presenting with inhalational anthrax in the United States received AIG-IV. Two of them survived, but no conclusion could be drawn out of these disparate records (8).

Eventually, a substantial amount of resources has been invested in the development of anthrax antitoxin antibodies, with no extensive assessment of their efficacy (6).

We discuss here the potential scientific reasons of the discrepancies observed between the manufacturer claims and the puzzling therapeutic effects observed in preclinical studies. We propose that use of the animal rule to grant the antibodies’ authorization was one of the causes of the misanalysis but not the only cause. Defective inductive reasoning may have been an additional cause of bias, as well as the absence of taking into account the singularity of each anthrax animal model.

## IS THE PA COMPONENT OF THE TOXINS THE JUDICIOUS THERAPEUTIC TARGET?

The pathophysiology of anthrax, caused by Bacillus anthracis, is complex and depends mainly on two critical virulence factors to proliferate in the host body: a tripartite toxin with two different activities and a poly-γ-d-glutamate capsule anchored to the cell wall (9). As a matter of fact, it should be kept in mind that anthrax is a toxi-infection (i.e., a combination of toxin and bacterial-sepsis components). The two toxins are made up of three secreted proteins, protective antigen (PA), edema factor (EF), and lethal factor (LF). Functionally, PA plays the central role as the unique component binding the toxin to the cell receptor. As a result, the combination of PA with EF or LF forms edema toxin (ET) or lethal toxin (LT), respectively. LF is a metalloprotease cleaving most of the mitogen-activated protein (MAP) kinase kinases and activating NLRP1B inflammasome in rodents through proteasome-mediated degradation of the N-terminal domains (10), while EF is a calmodulin-dependent adenylyl-cyclase (9). From the beginning, PA has been regarded from a conceptual standpoint as the most important immunological target because it is essential for the entry of both LF and EF. Interestingly, the discovery that the B. anthracis culture supernatant contains a “protective antigen” dates before the precise identification of the three toxin moieties in the 1950s by Harry Smith’s group (11). Antianthrax animal serum was used before the antibiotic era with some success, but it was also before the identification of the PA component (12). Thus, the serum targeted multiple proteins, and these data could not be conclusive. A wealth of further studies focusing on PA as an antigen led in the 1960s to the development of an acellular PA-based vaccine, establishing clearly the role of anti-PA immunity in the prevention of anthrax. The efficacy of murine anti-PA serum and antibodies was later demonstrated in guinea pigs with the fully virulent Ames strain (13), but anti-PA antibody studies substantially flourished after the 2001 anthrax letter attacks, with the help of recombinant monoclonal antibody technologies and with the pursuit of a new, improved PA-based vaccine (8). At the dawn of the 21st century, a race between several companies was virtually launched to develop anti-PA antibodies as an adjunct therapy for inhalational anthrax. More than six antibodies have been developed in the United States, and one has been developed in France, while two in the United States (raxibacumab and obiltoxaximab) and one in Europe (raxibacumab) have already been authorized. The choice of PA as a unique therapeutic target was not questioned until a recent meta-analysis of nine studies including 4 species and 748 animals revealed that all but one study failed to reach statistical significance (5). Subsequently, some called for investing money in additional research rather than stockpiling drugs with limited effects (6).

## REEXAMINING ANTI-PA ANTIBODY EFFECTS AND TOXIN PATHOGENESIS

**FIG 1:**
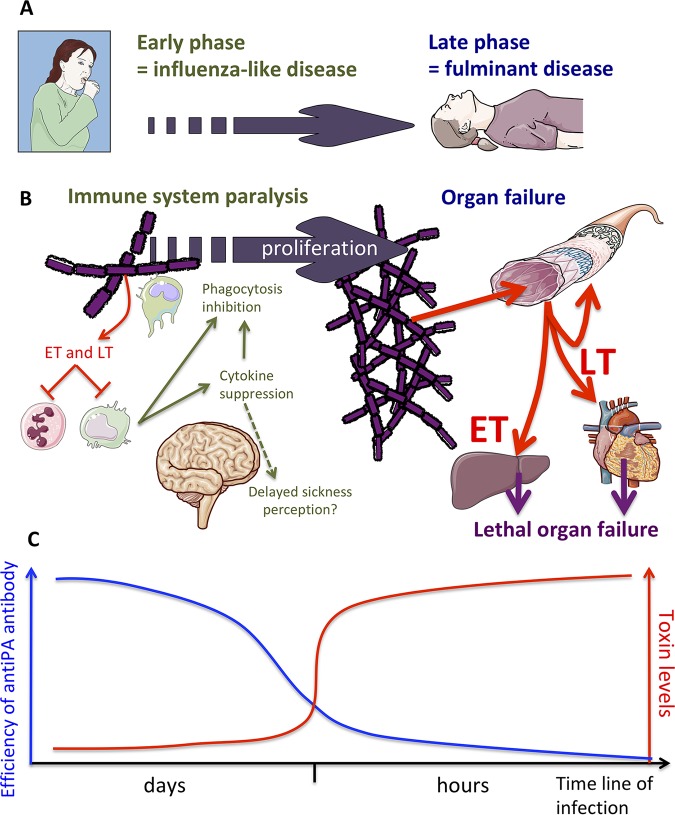
Effects of anthrax toxins at early and late phases of the infection. (A) The clinical presentation starts with an influenza-like illness that evolves to a fulminant disease, leading to death. (B) At the early stage of the infection, low levels of toxins (red arrows) acting locally to impair mainly immune cells (green arrows) are detected. Immune cells are suppressed, do not promote phagocytosis or pathogen elimination, and do not produce alarmins and pyrogenic cytokines, inducing a delay in the perception of sickness by the brain. In contrast, at the late stage of the infection, the disease is fulminant, with very high levels of circulating toxins (red arrows). At this stage, LT induces its toxicity to cardiomyocytes and vascular smooth muscles, while ET targets hepatocytes, leading to heart and liver failure (purple arrows). (C) Finally, anti-PA antibodies are efficient during prophylaxis and at the early stage of the infection, while their efficiency decreases with the increase in toxin level at the fulminant stage of infection, when patients are clinically symptomatic.

After inhalation of spores, the spores are captured by macrophages and dendritic cells, and transported to the draining lymph nodes, where germination occurs (14). Subsequent toxin release inhibits local immune cells (9, 14). Clinically, inhalational anthrax has an incubation period varying from 1 day to 6 weeks. The initial phase begins with nonspecific influenza-like symptoms lasting several days (malaise, myalgia, mild fever, and a nonproductive cough) (Fig. 1A). The fulminant stage follows with high fever, shock, and respiratory distress, leading to death typically within 24 to 36 h.

The role of toxins in the pathogenesis was suspected a long time ago, when Harry Smith showed that antibiotic treatment of guinea pigs would not save the animals once they reach the blood bacterial level of 10^6^ bacteria/ml (11). This was a strong indication that toxins played a crucial role *in vivo* (15). It took almost 4 decades to decrypt the biochemical activities of both toxins, as well as their complex paths of entry. Over the past decade, critical advances in recollecting biochemical data and animal models have been made by Moayeri et al. using genetic conditional ablation of the PA receptor (9). One critical point was the impairing effects of toxins on almost all components of the immune system, but their relevance *in vivo* in the early phase of the infection was eventually demonstrated by the conditional ablation of the PA receptor in myeloid cells of mice (9). In the absence of the toxin’s cellular effects on innate myeloid cells, the infection with an noncapsulated toxinogenic Ames strain is perfectly controlled, demonstrating that myeloid cells are crucial for infection control (Fig. 1B).

We propose here that an indirect significance of immune system freezing (as exemplified by the inhibition of pyretic cytokines) is that the immune system does not properly alert the brain. Neuro-immune communication is important for defense against infections (16). Numerous immune mediators (alarmins, cytokines) trigger neurogenic defense mechanisms, such as fever or sickness behavior, or modulate the behavior. The perception of a toxin-induced, global sickness may explain some clinical features of the disease. The silence of the immune system may partially explain why a life-threatening bacterial infection has a relatively mild, influenza-like clinical presentation. This point has important drawbacks, as shown by the fact that several patients who died in 2001 were withdrawn from the hospital with a wrong diagnosis, before being taken in charge too late, at the fulminant stage of disease (1).

A second point is how the toxin kills patients. The secret has partially been solved by conditional genetic ablation of the PA receptor in an elegant study demonstrating that LT kills by targeting cardiomyocytes and vascular smooth muscles, while ET targets hepatocytes (17).

Recent data describe circulating levels of toxins in mice (18) and nonhuman primates (NHP) (19). This point is of paramount physiological importance, as toxin levels condition the evolution of anthrax. The latter study, an inhalational anthrax model with macaques, showed a dramatic increase of LT at the terminal stage of the infection (19). At this stage, when animals present clinical signs, saturating levels of toxins further saturate the tissues, and anti-PA antibody may arrive too late. Toxins are probably already inside cells, and anti-PA antibody cannot access their targets. In fact, there is a race against time between anti-PA and PA (and toxin formation plus cell entry), as clearly suggested by the fact that anti-PA preexposure prophylaxis is highly efficient, while postexposure efficiency decreases dramatically over time (Fig. 1C). As an example, obiltoxaximab at the dose of 16 mg/kg of body weight protected 100% of macaques in a prechallenge administration, while protection dropped to 93%, 43%, and 25% when the antibody was administered with a delay of 24, 36, and 48 h, respectively (20).

## WHAT WENT WRONG WITH ANTI-PA ANTIBODY EVALUATION?

### Inductive bias.

First, we think that an inductive bias may have led to inadequate analysis of the results. The confidence in therapeutics targeting PA may have been overrated by the long-known and accepted efficiency of a PA-based vaccine in animal models. This may have resulted in defective inductive reasoning: if anti-PA vaccines are protective and anti-PA antibodies confer a protection in prophylaxis, anti-PA antibodies must protect in postexposition settings (21). Results that do not fit with the model of protection are neglected or assimilated as nonsignificant for the whole analysis. As a paradigm of this inductive bias, the study claiming an added benefit of the raxibacumab and levofloxacin combination over levofloxacin showed that only 32/39 (82%) rabbits survived an Ames challenge with the raxibacumab and levofloxacin combination, versus 24/37 (65%) of rabbits given levofloxacin only (22). However, the difference in survival rate between the two groups did not reach statistical significance (*P* = 0.0874), ruling out a benefit of the combination upon the employment of proper statistical standards, although the authors claimed a “higher survival rate” (22).

### Animal model bias.

**FIG 2:**
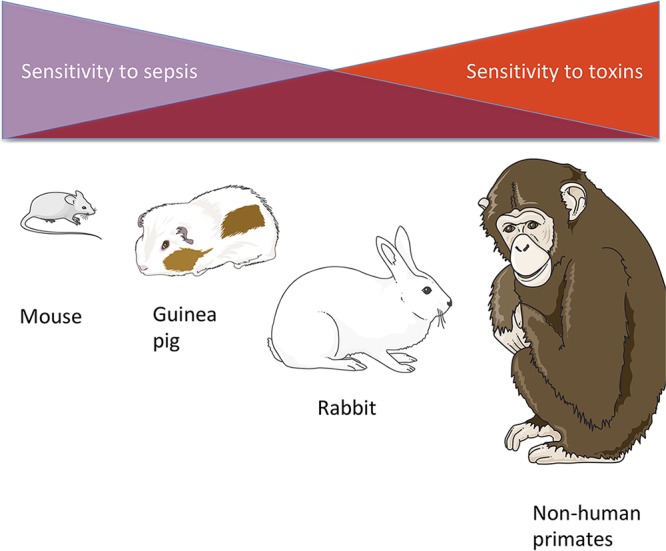
Sensitivities of animal models to toxins. The sensitivities of animal models to anthrax toxins differ among species. Mouse is the most resistant to toxins, as it cannot be protected by a PA-based vaccine against a virulent Bacillus anthracis strain. Guinea pig is said to be intermediate, while rabbit and NHP are the most sensitive to toxins.

Second, we stress that anthrax animal models are biased *per se* because anthrax is a toxi-infection, and models balance between both facets of the disease (23). Each species differs in the way it reflects the human sensitivity to toxin prevalence and bacillus proliferation, but these factors have not been properly accounted for in the analysis. The animal rule states that the effect must be demonstrated in more than one animal species before it can be expected to become predictive for humans. Raxibacumab has been the first therapeutic officially accepted by this means. The variety of species’ sensitivities to toxins may have skewed the assessment of the anti-PA antibodies. Different species react differently to the different facets of anthrax. The most commonly used species are mouse, guinea pig, rabbit, and NHP. However, they differ greatly in their sensitivities to toxemia. Mice (except immunocompromised A/J mice infected by the Sterne strain) are more sensitive to the infection, while guinea pigs and rabbits are more sensitive to the toxins (Fig. 2). As a result, most anti-PA antibodies have been evaluated first in guinea pig and rabbit models. NHP are phylogenetically closer to humans but are more expensive, and their use is limited by ethics considerations. Furthermore, rats are also phylogenetically close to mice but react quite differently to anthrax infection (they are resistant). Finally, many studies have been found to be too limited in size to reach statistical significance. As NHP is also reputed to be sensitive to toxins, it is generally said that rabbit is a good model for human. In fact, rabbit has been widely used for assessing vaccine efficacy. As a result, all anti-PA antibodies have been evaluated mostly with rabbit and NHP. In fact, we think that rabbit has many limitations because of its obvious weak relationship to human. Moreover, in a model of intravenous injection of Ames strain bacilli, rabbit is equally sensitive to LT and ET (24) while NHP is more sensitive to LT (25), suggesting divergences of both models in toxin sensitivity. It is interesting to notice that in these models of intravenous injection, the animal infected with the wild-type strain ended with a significantly higher load of bacteria in the spleen, suggesting indirectly that toxins act in these models as immune suppressors (24, 25).

Third, in all study settings, animals are infected and monitored up to the endpoint, which is death, but so far no study has included life support or hemodynamic support, which is part of the treatment in humans. Interestingly, in a model of intoxination (without infection), anti-PA antibodies may improve outcomes during shock with ET alone or together with LT in canines if antibody is not delayed beyond 6 h (26). Hemodynamic support alone does not improve survival. This suggests that further study design should include hemodynamic support.

## WHERE TO GO NOW?

Even if there may be a benefit of anti-PA antibodies at the early anthrax stage, further studies are urgently needed to establish the advantage along the course of infection in NHP models with sufficient numbers of animals. These studies should also try to be as realistic as possible to the management of patients and should include also hemodynamic support, especially for late-stage-infection experiments. A clearer consensus should be established around what evidence should be proven in any anthrax model. Eventually, these data may also show that there may be a restricted utility of anti-PA antibodies based on toxin levels and timing.

Moreover, at some point, anti-PA antibodies may no be longer efficient because a sufficient level of toxins has already penetrated the cells. The level of toxin may have triggered a point of no return. Other strategies, such as small-molecule inhibitors of toxins, as well as antibodies targeting other components of the disease, such as sepsis, should be developed.

## References

[1] Jernigan JA, Stephens DS, Ashford DA, Omenaca C, Topiel MS, Galbraith M, Tapper M, Fisk TL, Zaki S, Popovic T, Meyer RF, Quinn CP, Harper SA, Fridkin SK, Sejvar JJ, Shepard CW, McConnell M, Guarner J, Shieh WJ, Malecki JM, Gerberding JL, Hughes JM, Perkins BA, Anthrax Bioterrorism Investigation T. 2001 Bioterrorism-related inhalational anthrax: the first 10 cases reported in the United States. Emerg Infect Dis 7:933–944. doi:10.3201/eid0706.010604. 11747719 PMC2631903

[2] Holty JE, Bravata DM, Liu H, Olshen RA, McDonald KM, Owens DK 2006 Systematic review: a century of inhalational anthrax cases from 1900 to 2005. Ann Intern Med 144:270–280. doi:10.7326/0003-4819-144-4-200602210-00009. 16490913

[3] Project BioShield Act. 2004 Public law 108-276. 108th Congress, Washington, DC https://www.congress.gov/108/plaws/publ276/PLAW-108publ276.pdf.

[4] Larsen JC, Disbrow GL 2017 Project BioShield and the Biomedical Advanced Research Development Authority: a ten year progress report on meeting U.S. preparedness objectives for threat agents. Clin Infect Dis 64:1430–1434. doi:10.1093/cid/cix097. 28158662

[5] Xu W, Ohanjanian L, Sun J, Cui X, Suffredini D, Li Y, Welsh J, Eichacker PQ 2017 A systematic review and meta-analysis of preclinical trials testing anti-toxin therapies for B. anthracis infection: a need for more robust study designs and results. PLoS One 12:e0182879. doi:10.1371/journal.pone.0182879. 29200425 PMC5714336

[6] Vietri NJ 2018 Does anthrax antitoxin therapy have a role in the treatment of inhalational anthrax? Curr Opin Infect Dis 31:257–262. doi:10.1097/QCO.0000000000000446. 29570493

[7] Cui X, Nolen LD, Sun J, Booth M, Donaldson L, Quinn CP, Boyer AE, Hendricks K, Shadomy S, Bothma P, Judd O, McConnell P, Bower WA, Eichacker PQ 2017 Analysis of anthrax immune globulin intravenous with antimicrobial treatment in injection drug users, Scotland, 2009–2010. Emerg Infect Dis 23:56–65. doi:10.3201/eid2301.160608. 27983504 PMC5176236

[8] Huang E, Pillai SK, Bower WA, Hendricks KA, Guarnizo JT, Hoyle JD, Gorman SE, Boyer AE, Quinn CP, Meaney-Delman D 2015 Antitoxin treatment of inhalation anthrax: a systematic review. Health Secur 13:365–377. doi:10.1089/hs.2015.0032. 26690378 PMC4710135

[9] Moayeri M, Leppla SH, Vrentas C, Pomerantsev AP, Liu S 2015 Anthrax pathogenesis. Annu Rev Microbiol 69:185–208. doi:10.1146/annurev-micro-091014-104523. 26195305

[10] Sandstrom A, Mitchell PS, Goers L, Mu EW, Lesser CF, Vance RE 2019 Functional degradation: a mechanism of NLRP1 inflammasome activation by diverse pathogen enzymes. Science 364:3aau1330. doi:10.1126/science.aau1330. PMC6532986 30872533

[11] Smith H 2000 Discovery of the anthrax toxin: the beginning of in vivo studies on pathogenic bacteria. Trends Microbiol 8:199–200. doi:10.1016/S0966-842X(00)01755-8. 10785632

[12] Lockwood CB, Andrewes FW 1905 A case of cutaneous anthrax successfully treated by Sclavo's serum. Br Med J 1:16. doi:10.1136/bmj.1.2297.16. PMC2318710 20761854

[13] Little SF, Ivins BE, Fellows PF, Friedlander AM 1997 Passive protection by polyclonal antibodies against Bacillus anthracis infection in guinea pigs. Infect Immun 65:5171–5175. 9393812 10.1128/iai.65.12.5171-5175.1997 PMC175745

[14] Goossens PL, Tournier JN 2015 Crossing of the epithelial barriers by Bacillus anthracis: the known and the unknown. Front Microbiol 6:1122. doi:10.3389/fmicb.2015.01122. 26500645 PMC4598578

[15] Smith H, Keppie J 1954 Observations on experimental anthrax; demonstration of a specific lethal factor produced in vivo by Bacillus anthracis. Nature 173:869–870. doi:10.1038/173869a0. 13165673

[16] Kipnis J 2018 Immune system: the “seventh sense.” J Exp Med 215:397–398. doi:10.1084/jem.20172295. 29339443 PMC5789422

[17] Liu S, Zhang Y, Moayeri M, Liu J, Crown D, Fattah RJ, Wein AN, Yu ZX, Finkel T, Leppla SH 2013 Key tissue targets responsible for anthrax-toxin-induced lethality. Nature 501:63–68. doi:10.1038/nature12510. 23995686 PMC4080305

[18] Rougeaux C, Becher F, Ezan E, Tournier JN, Goossens PL 2016 In vivo dynamics of active edema and lethal factors during anthrax. Sci Rep 6:23346. doi:10.1038/srep23346. 26996161 PMC4800402

[19] Boyer AE, Quinn CP, Hoffmaster AR, Kozel TR, Saile E, Marston CK, Percival A, Plikaytis BD, Woolfitt AR, Gallegos M, Sabourin P, McWilliams LG, Pirkle JL, Barr JR 2009 Kinetics of lethal factor and poly-d-glutamic acid antigenemia during inhalation anthrax in rhesus macaques. Infect Immun 77:3432–3441. doi:10.1128/IAI.00346-09. 19506008 PMC2715684

[20] Yamamoto BJ, Shadiack AM, Carpenter S, Sanford D, Henning LN, Gonzales N, O'Connor E, Casey LS, Serbina NV 2016 Obiltoxaximab prevents disseminated Bacillus anthracis infection and improves survival during pre- and postexposure prophylaxis in animal models of inhalational anthrax. Antimicrob Agents Chemother 60:5796–5805. doi:10.1128/AAC.01102-16. 27431219 PMC5038297

[21] Chalmers AF 1976 What is the thing called science? An assessment of the nature and status of science and its methods. University of Queensland Press, Saint Lucia, Australia.

[22] Migone TS, Bolmer S, Zhong J, Corey A, Vasconcelos D, Buccellato M, Meister G 2015 Added benefit of raxibacumab to antibiotic treatment of inhalational anthrax. Antimicrob Agents Chemother 59:1145–1151. doi:10.1128/AAC.04606-14. 25487792 PMC4335881

[23] Goossens PL 2009 Animal models of human anthrax: the quest for the Holy Grail. Mol Aspects Med 30:467–480. doi:10.1016/j.mam.2009.07.005. 19665473

[24] Lovchik JA, Drysdale M, Koehler TM, Hutt JA, Lyons CR 2012 Expression of either lethal toxin or edema toxin by Bacillus anthracis is sufficient for virulence in a rabbit model of inhalational anthrax. Infect Immun 80:2414–2425. doi:10.1128/IAI.06340-11. 22526673 PMC3416453

[25] Hutt JA, Lovchik JA, Drysdale M, Sherwood RL, Brasel T, Lipscomb MF, Lyons CR 2014 Lethal factor, but not edema factor, is required to cause fatal anthrax in cynomolgus macaques after pulmonary spore challenge. Am J Pathol 184:3205–3216. doi:10.1016/j.ajpath.2014.08.008. 25285720 PMC4258508

[26] Remy KE, Cui X, Li Y, Sun J, Solomon SB, Fitz Y, Barochia AV, Al-Hamad M, Moayeri M, Leppla SH, Eichacker PQ 2015 Raxibacumab augments hemodynamic support and improves outcomes during shock with B. anthracis edema toxin alone or together with lethal toxin in canines. Intensive Care Med Exp 3:9. doi:10.1186/s40635-015-0043-4. 26097803 PMC4473792

